# Optical-CT 3D Dosimetry Using Fresnel Lenses with Minimal Refractive-Index Matching Fluid

**DOI:** 10.1371/journal.pone.0152606

**Published:** 2016-03-28

**Authors:** Steven Bache, Javian Malcolm, John Adamovics, Mark Oldham

**Affiliations:** 1 Duke University Medical Physics Graduate Program, Durham, NC, 27705, United States of America; 2 Department of Chemistry, Rider University, Lawrenceville, NJ, 08648, United States of America; 3 Department of Radiation Oncology, Duke University Medical Center, Durham, NC, 27710, United States of America; North Shore Long Island Jewish Health System, UNITED STATES

## Abstract

Telecentric optical computed tomography (optical-CT) is a state-of-the-art method for visualizing and quantifying 3-dimensional dose distributions in radiochromic dosimeters. In this work a prototype telecentric system (DFOS—Duke Fresnel Optical-CT Scanner) is evaluated which incorporates two substantial design changes: the use of Fresnel lenses (reducing lens costs from $10-30K t0 $1-3K) and the use of a ‘solid tank’ (which reduces noise, and the volume of refractively matched fluid from 1ltr to 10cc). The efficacy of DFOS was evaluated by direct comparison against commissioned scanners in our lab. Measured dose distributions from all systems were compared against the predicted dose distributions from a commissioned treatment planning system (TPS). Three treatment plans were investigated including a simple four-field box treatment, a multiple small field delivery, and a complex IMRT treatment. Dosimeters were imaged within 2h post irradiation, using consistent scanning techniques (360 projections acquired at 1 degree intervals, reconstruction at 2mm). DFOS efficacy was evaluated through inspection of dose line-profiles, and 2D and 3D dose and gamma maps. DFOS/TPS gamma pass rates with 3%/3mm dose difference/distance-to-agreement criteria ranged from 89.3% to 92.2%, compared to from 95.6% to 99.0% obtained with the commissioned system. The 3D gamma pass rate between the commissioned system and DFOS was 98.2%. The typical noise rates in DFOS reconstructions were up to 3%, compared to under 2% for the commissioned system. In conclusion, while the introduction of a solid tank proved advantageous with regards to cost and convenience, further work is required to improve the image quality and dose reconstruction accuracy of the new DFOS optical-CT system.

## Introduction

Optical computed tomography (optical-CT) is a technique for imaging 3D dose distributions in radiochromic and gel dosimeters [1,2] It is the visible light analog of X-ray CT in that linear attenuation coefficients of visible light are reconstructed throughout a partially transparent dosimeter. Optical-CT was originally used in the context of polymer gel dosimeters, which exhibit radiation-induced polymerization, changing the optical density (OD) of the gel [3,4,5]. Optical-CT has also been used as an alternative to MRI-readout of Fricke dosimeters, which exhibit a dose-dependent oxidation of Fe^2+^ ions, resulting in an optical density change [6–9]. Radiochromic plastic dosimeters such as Presage (PRESAGE®) (Hueris, Skillman, NJ) have been well documented [10–14] and have been shown to exhibit a linear dose response, water/tissue equivalency, insensitivity to ambient light and temperature, and require no external case or mold, allowing a variety of physical alterations. These characteristics make radiochromic dosimeters valuable for diverse applications including the verification of complex radiation treatment therapies and validation of deformable image/dose registration algorithms (see summary in Oldham 2014 [15]). The highly transparent polyurethane matrix of Presage, coupled with the light absorbing nature of the radiochromic dye (Malachite Green analog), renders Presage amenable to accurate optical-CT dosimetry by virtue of the very low-level of contaminant stray light.

The DLOS (Duke large Optical-CT Scanner) was recently commissioned for clinical 3D dosimetry [16] and represents a state-of-the-art 3D dosimetry system when used with Presage. The DLOS system ensures a strict parallel ray-geometry through the use of a precision telecentric light source generating a collimated parallel beam which is incident normally to a square fluid-bath containing the dosimeter to be imaged immersed in a refractively matched fluid. Telecentric lenses must be at least as big as the maximum dimension of the dosimeter to be imaged. To enable imaging of a sizeable dosimeter (e.g. head-sized) very large telecentric lenses are required which dramatically increases the cost of the DLOS system. The utilization of a fluid bath is an essential feature of telecentric and many other optical-CT systems to date [1,9,17]. The volume of refractive index (RI) matching fluid in the DLOS fluid bath is 5 liters, which introduces a level of expense and inconvenience in terms of keeping the fluid clean, and in making fine-tune adjustments for dosimeters of different RI. Several authors have investigated the feasibility of omitting or reducing the refractive index matching fluid [18–21] as well as the incorporation of Fresnel lenses [22,23]. These same concerns apply to the DMOS (Duke Mid-sized Optical-CT Scanner) system which is a scaled down version of DLOS with maximum field of view of 12cm [24].

In this work, we explore the feasibility of accurate 3D dosimetry with the DFOS scanner incorporating two innovations: the solid tank which reduces the amount of RI matching fluid by 98%; and the utilization of Fresnel lenses.

## Materials and Methods

The efficacy of 3D dosimetry capability with the Fresnel and tank-based DFOS system was investigated by direct comparison against existing optical-CT systems and predicted dose distributions from a commissioned treatment planning system (TPS) on several irradiated cylindrical Presage 3D dosimeters. The following three sections describe the details of the DFOS system modifications and operation, details of the irradiations, and the methods of dosimetry analysis, respectively.

### DFOS modifications

The DFOS system replaces the convex telecentric lenses used in both the DLOS and DMOS with a Fresnel lens system (Light Works, Toledo, OH). This results in cost savings from about $10-30K to $1-3K for the lens system alone, as well as a lens weight reduction from around 7 kg to 500 g. The DFOS also features a solid polyurethane tank in place of the large fluid bath for refractive index-matching fluid utilized by the DLOS/DMOS. The two major modifications made to the DFOS–the Fresnel lenses and polyurethane tank–are detailed below.

#### Fresnel lenses

**Fig 1:**
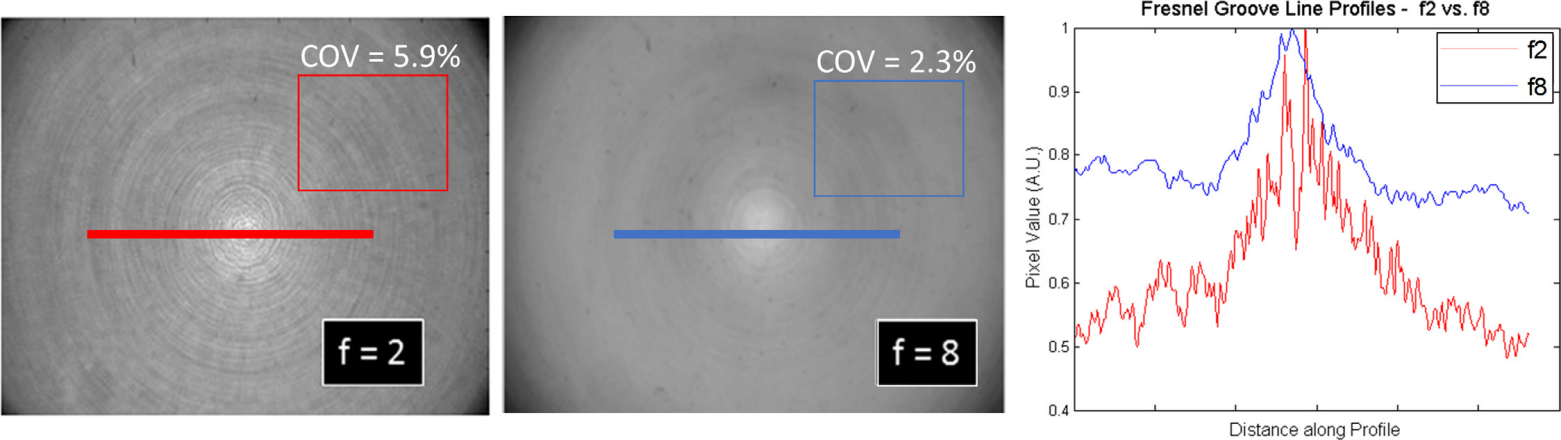
Fresnel artifacts. Non-uniformities caused by Fresnel grooves (left) may be reduced by increasing the f-stop (right) at the imaging lens. Remaining non-uniformities are corrected by acquiring pre- and post-irradiation images.

Fresnel lenses were developed for applications requiring a lighter weight and less expense. Fresnel lenses have inherent non-uniformity due to their grooved structure which can cause a series of concentric circles called the Fresnel effect. In addition, slight misalignment of the Fresnel grooves in the two Fresnel lenses may cause a Moiré effect, an inconvenience not present in standard convex telecentric systems. These effects are reduced by carefully tuning the focal point of the system to lie directly between the pair of lenses, and also by simultaneously decreasing the imaging aperture (increasing the f-number,) while increasing the source spot size (LED output aperture). This effect is illustrated in Fig 1 which shows DFOS flood field images acquired with different f-stop settings. The Fresnel grooves are reduced but still visible, however this structure can be effectively eliminated as a source of noise and inaccuracy in optical-CT through the use of flood field corrections and the acquisition of pre and post irradiation images [16].

#### Solid tank

The second modification present in the DFOS is the solid polyurethane tank. For the DFOS system, the glass aquarium was replaced with a 17x17x17 cm^3^ polyurethane cube with a cylindrical hollow bore of diameter 11.5cm and height 10cm, allowing for placement of a standard 1kg 11cm diameter x 10cm dosimeter. The tank was constructed from the same polyurethane material as Presage dosimeters. The 2.5mm air gap surrounding the dosimeter requires ~100 mL of fluid of match the refractive index between the polyurethane tank (RI = 1.5) to Presage dosimeters (RI = 1.47–1.51), compared to ~14 liters of fluid for the DLOS system.

#### DFOS optical-CT acquisition

DFOS optical-CT requires acquisition of projection images acquired both pre- and post-irradiation in order to reconstruct images of the change in optical density (OD). Incident and exiting intensities are “dark” corrected to account for any inherent noise in the imaging CCD array, leading to a corrected definition of OD:

$$OD=-\log_{10}\;(\frac{I-dark}{I_{0}-dark})=-\log_{10}\;\left(\frac{I^{\prime }}{{I\prime }_{0}}\right)$$ (1)

Therefore, OD change (ΔOD) in each voxel within the dosimeter–the quantity directly proportional to absorbed dose–can be calculated as:

$$\Delta OD={OD}_{post}-{OD}_{pre}=\log_{10}\;(\frac{{I\prime }_{pre}}{{I\prime }_{post}})-\log_{10}\;\left(\frac{{I\prime }_{0,pre}}{{I\prime }_{0,post}}\right)$$ (2)

An incident light image I_0_ or “flood-field” may be acquired by filling the tank with RI fluid matched to the polyurethane tank and dosimeter. However this would be inconvenient, requiring a filling of the solid-tank bore with RI fluid for the flood field acquisition, followed by removal of almost all the fluid when the dosimeter is to be scanned. We note that the stability of the solid tank (i.e. the constancy of the locations of any impurity specs and low frequency background attenuation), may enable determination of ΔOD from a simplified form of Eq 2, shown in Eq 3.

$$\Delta OD=\log_{10}\;(\frac{{I\prime }_{pre}}{{I\prime }_{post}}).$$ (3)

**Fig 2:**
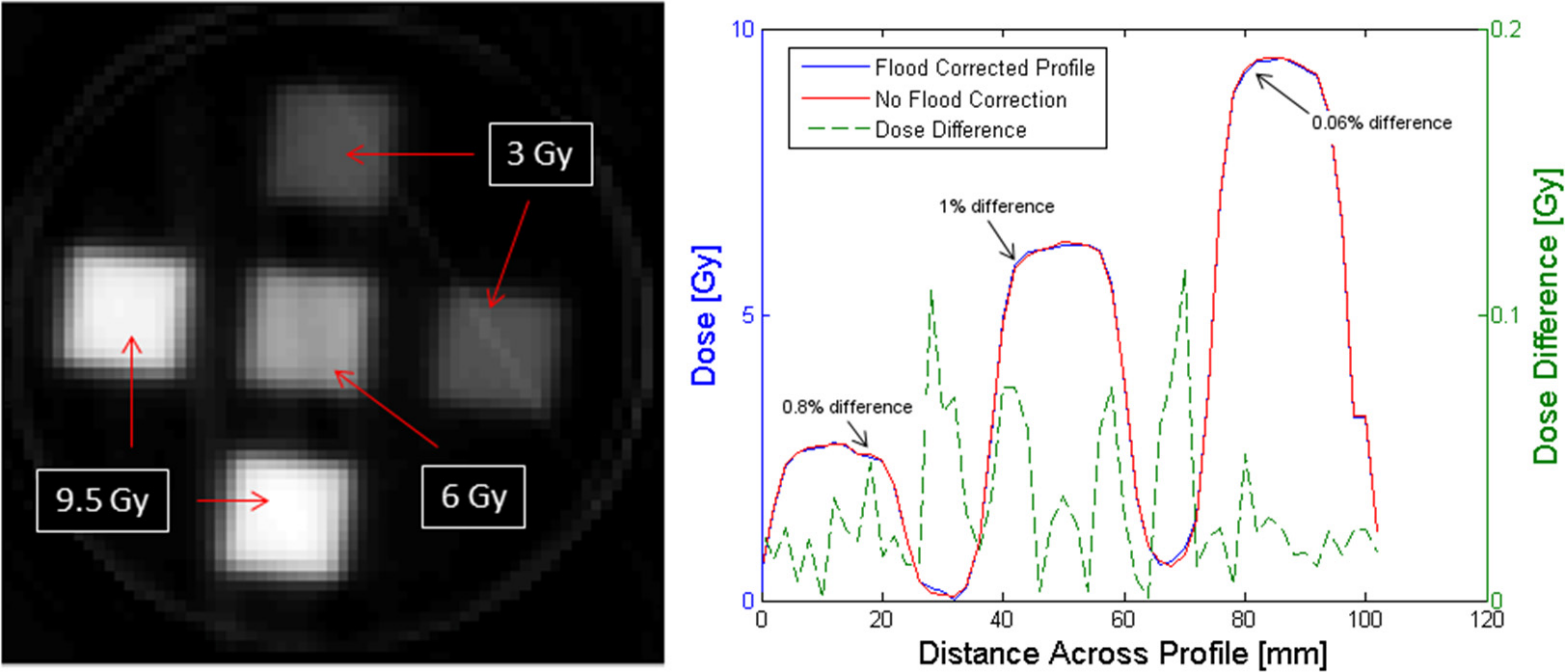
Flood Field Omission. The stability of the solid-tank enables a simplified acquisition technique without requiring a flood field. (a) Reconstructed central axial slice through a dosimeter irradiated with 5 small fields incident on the upper surface with different doses. (b) Reconstructing the data with and without flood-field correction (techniques outlined in previous section) indicate the new flood-field-free reconstructions were equivalent to the conventional method.

Eq 3 enables ΔOD to be determined directly from a pre- and post-irradiation scan-pair, without the need for any flood field measurement. This approach is valid only if the flood fields are equivalent in the two scans, such that the entire imaging chain, including the solid tank and fluid, as well as LED-output remain unchanged between pre- and post- irradiation acquisitions. The validity of this approach was investigated by application to a dosimeter irradiated with a simple 5-field anterior-posterior treatment (Fig 2). Pre- and post-irradiation optical-CT data was acquired with DLOS, and the 3D dose distribution reconstructed conventionally (Eq 2) and with the new flood field free technique (Eq 3).

### Treatment Plans

Radiation treatments were given to three 11cm x 10cm (height x diameter) Presage radiochromic dosimeters for subsequent scanning. For this work, the same Presage formulation was used for all dosimeters, which consists of a polyurethane matrix, 1.5% leuco-dye, 0.5% carbon tetrabromide, and (cyclohexanone) solvent. For all treatments, CT scans were taken of the dosimeter and imported to the Eclipse (Varian Medical Systems, Palo Alto, CA) treatment planning software (TPS) for dose prediction for each treatment. Absorbed dose from the CT scan was assumed to be negligible (on the order of 1–2 mGy) compared to the prescribed treatment doses for each plan. Dose was delivered by 6MV beams at a dose rate of 600 MU/min for all irradiations.

#### Four-field box treatments

**Fig 3:**
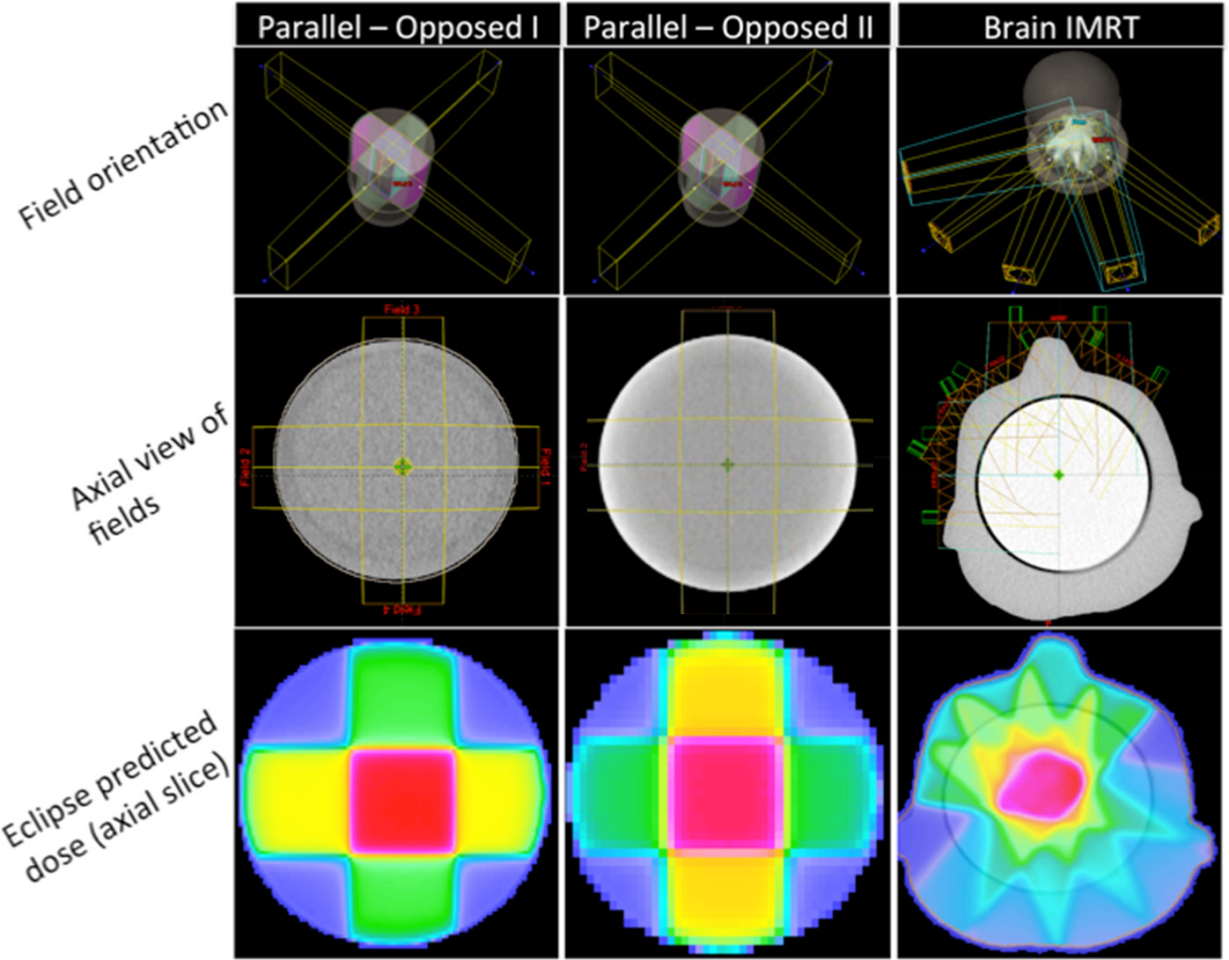
Treatment Plans. Four-field box treatments (left and center) and five-field brain IMRT (right) treatment plans from Eclipse TPS. View of IMRT axial view and predicted dose view scaled to show plastic head-insert.

Two four-field box 6MV treatment plans consisting of 4 beams each of field size 4cm x 10cm was created in Eclipse. For the first treatment, the lateral beams each delivered 500MU, and the vertical beams each delivered 250MU to give a maximum prescription dose of 1200cGy. This arrangement led to 3 distinct uniform calculated dose regions of 30%, 50% and 100% of the prescription dose (Fig 3). The second treatment was identical to the first but with prescription dose halved. The treatments were delivered axially to two separate dosimeters of similar size (11cm diameter, 10cm length) but from different batches of the same formulation, and at different times (separated several months apart). For the first irradiation the dosimeter was scanned in the DFOS scanner only, while the second dosimeter treated was scanned in all 3 scanners (DFOS, DMOS and DLOS). The previously commissioned and benchmarked DLOS system was considered the gold-standard optical-CT system.

#### Brain IMRT

The final irradiation was performed on a third cylindrical 11cm diameter x 10cm Presage dosimeter. Treatment consisted of a 5-field brain IMRT plan with a prescription dose of 500cGy to be delivered by 6MV photons to the cylindrical dosimeter through a plastic tissue-equivalent head phantom (Fig 3). The dosimeter was scanned with both the gold-standard DLOS and DFOS optical-CT system. While the 4-field box irradiation dose prescriptions were chosen for simplicity, this IMRT plan was taken directly from a patient plan at our institution.

### Data analysis

Each 3D dose distribution was obtained by acquiring 360 projection images in 1-degree increments both pre- and post-irradiation. Optical projection images were reconstructed with a custom MATLAB GUI (The Math Works, Natick, MA, USA). All reconstructions were made with a filtered back-projection algorithm utilizing the default MATLAB “iradon” function. All dose distributions were made with 2mm voxels and using the same Ram-Lak apodizing filter, so reconstructions were both scanner- and dose-independent. Quantitative comparison between optical-CT reconstructions and the corresponding treatment plan were made by computing the gamma passing rate at a threshold of 3%/3mm with respect to TPS predicted dose distribution [25]. For this preliminary study, relative dose was converted to absolute dose utilizing a point of known dose taken from the Eclipse predicted dose distribution and scaling the optical-CT reconstructed OD-change accordingly. This step is feasible for the purpose of investigating scanner performance because of the well-established linear dose response of Presage and the simplicity of these treatment plans [13,16,26].

DFOS 3D dose distributions were compared against the predicted dose distribution from the treatment planning system, and to corresponding DLOS and DMOS data. Dose agreement was evaluated through 3D gamma maps calculated with the Computational Environment for Radiotherapy Research (CERR) MATLAB code. Gamma criteria of 3%, 3mm were utilized with a 10% threshold. Before gamma calculation, each reconstructed dose distribution was manually registered to the Eclipse predicted dose distribution. This was accomplished by matching the orientations of the dosimeter edges within each distribution, as well as matching small holes drilled into the dosimeter suface which served as fiducial markers. A secondary check for proper registration was performed by qualitatively comparing line profiles through the distributions to be compared. Relative dose distributions in units of optical density change (ΔOD) per voxel were normalized to the maximum dose in the uniform region of the Eclipse distribution, a valid scaling due to the direct linear relationship between dose and ΔOD in Presage dosimeters [10]. Line profiles were drawn in the same axial slice across the center of each DFOS/DMOS/DLOS/Eclipse distribution. The ends of the dosimeter were cropped to avoid edge artifacts causing artificially enhanced dose differences in the gamma calculation.

## Results and Discussion

### DFOS dose accuracy

#### First 4 field box irradiation

**Fig 4:**
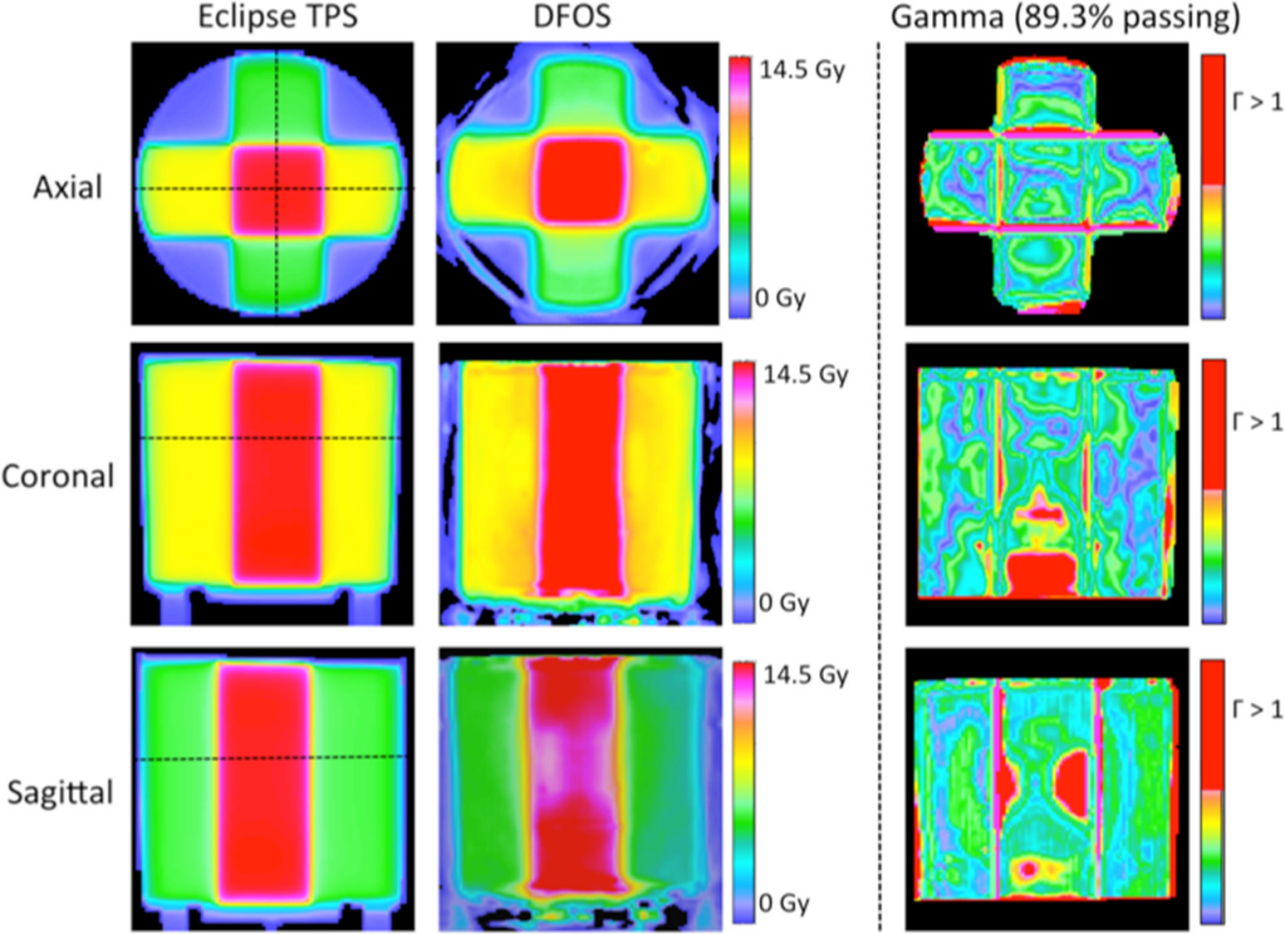
Treatment Plan 1—Results and Gamma Maps. Eclipse predicted dose (left), DFOS reconstructed dose (middle) and 3%/3mm gamma maps (right) for three orthogonal planes in parallel-opposed treatment plan I.

Fig 4 shows orthogonal slices through the TPS predicted and DFOS measured dose distribution of the first 4 field box irradiation, along with corresponding gamma maps. 3D gamma pass rates were 89.3% with a 3%/3mm criteria compared to Eclipse TPS prediction. The essential features of the predicted and measured dose distributions agree well qualitatively, but the gamma pass rate is substantially lower than desired. Inspection of the gamma plots reveals that most failing regions coincided with sharp dose gradients at the edges of differing dose regions. This feature of the DFOS images (seen in later images) appear to indicate an apparent penumbral blurring that could be the result of registration error in the manual alignment of the measured and calculated dose distributions. In addition, a region of non-uniformity was seen in the sagittal plane of the DFOS dose reconstruction, confined to the central slices of the reconstructed dose volume. Non-uniformities of this magnitude were not seen in other dosimeters (data shown below) suggesting the possibility of small imperfections in this dosimeter (e.g. non-uniformity of dose-response caused by inadequate mixing).

#### Second 4 field box irradiation

**Fig 5:**
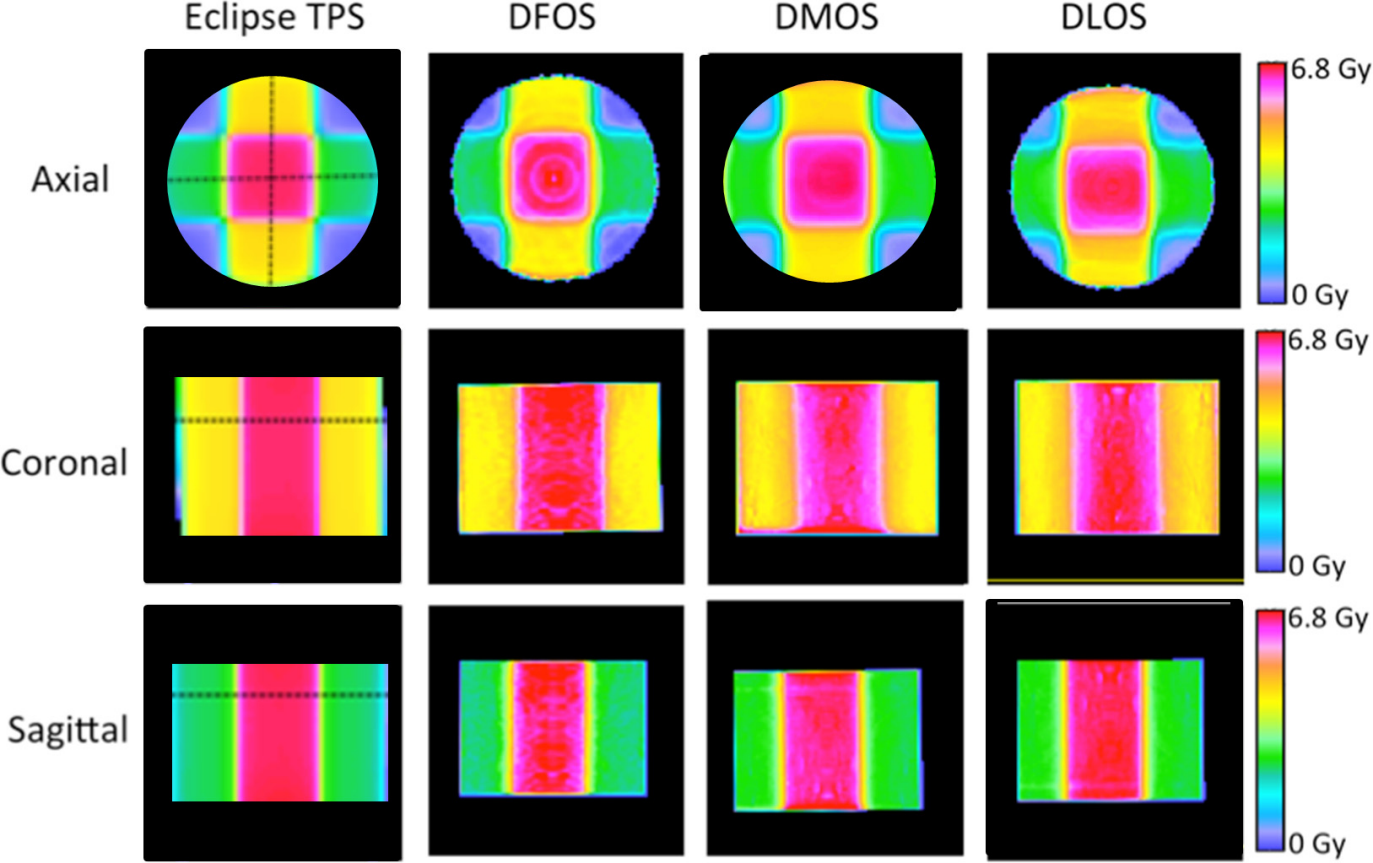
Treatment Plan 2—Results. Eclipse predicted dose (left), DFOS reconstructed dose (second column), DMOS reconstructed dose (third column), and DLOS reconstructed dose (right) for three orthogonal planes in four-field box irradiation.

**Fig 6:**
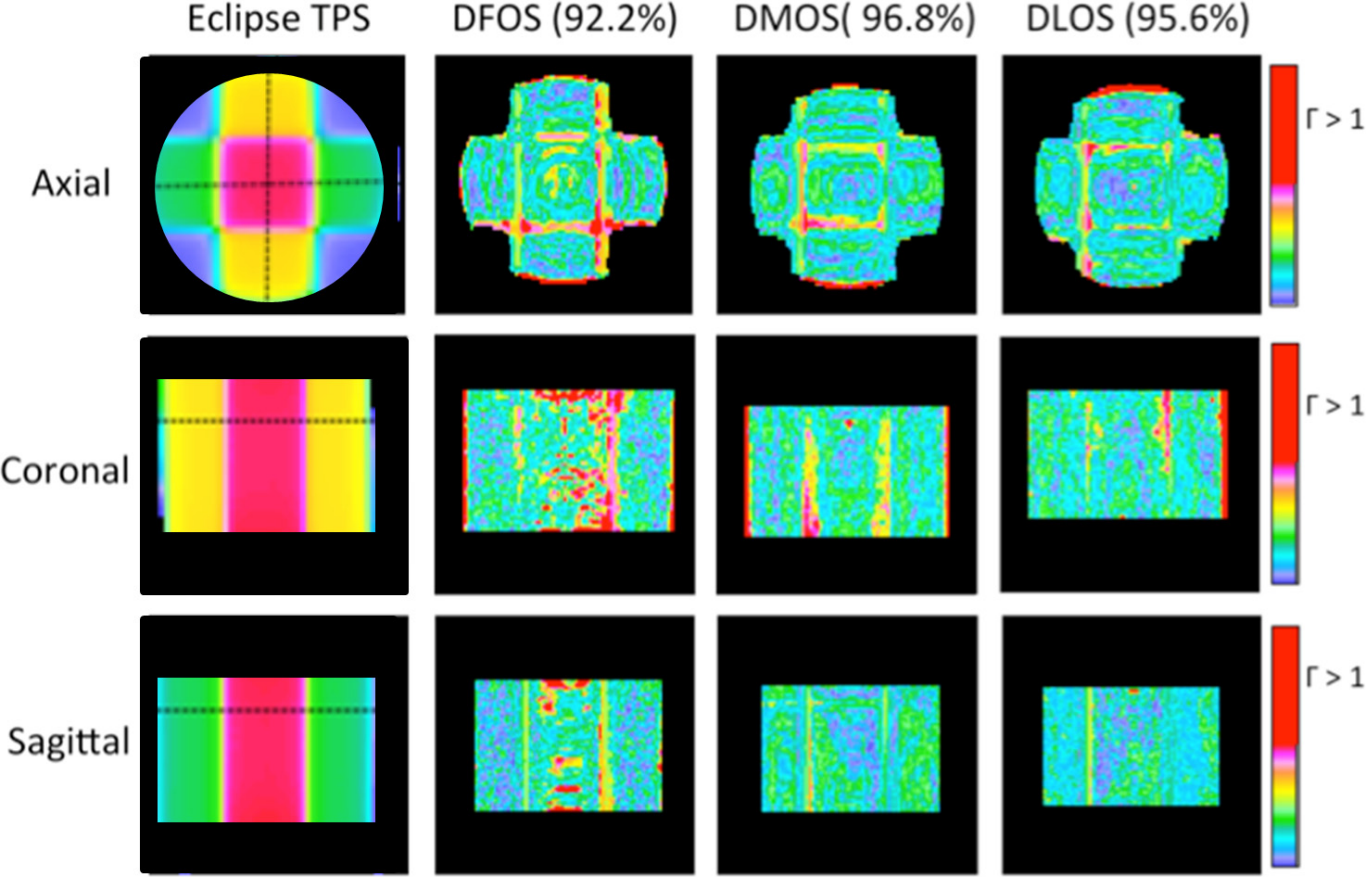
Treatment Plan 2—Gamma Maps. Three orthogonal views of 3%/3mm gamma maps for DFOS (column 2), DMOS (column 3), and DLOS (right) systems, with passing rates. Eclipse dose views (left) for reference.

For the second 4 field box irradiation, the DFOS, DLOS, and DMOS measured dose distributions were all available for comparison with Eclipse TPS prediction. Fig 5 and Fig 6 show slices through the reconstructed dose distributions and gamma maps, respectively. Qualitative comparison of the orthogonal dose-maps shows consistency of the main features of the dose distributions, although some pronounced ring artifacts are apparent in the DFOS images, and to a lesser extent in the DMOS. The 3D gamma pass rates for the DFOS, DLOS, and DMOS were 92.2%, 95.6%, and 96.8%, respectively, compared to Eclipse prediction. The DLOS and DMOS dose distributions had excellent agreement compared to each other (gamma pass rate of 99.0%), indicating good consistency between the two gold-standard scanners. The 3D gamma pass rate between the DLOS and DFOS systems was 98.2, slightly better than the DFOS/DMOS gamma pass rate of 97.9%.

#### Brain IMRT

**Fig 7:**
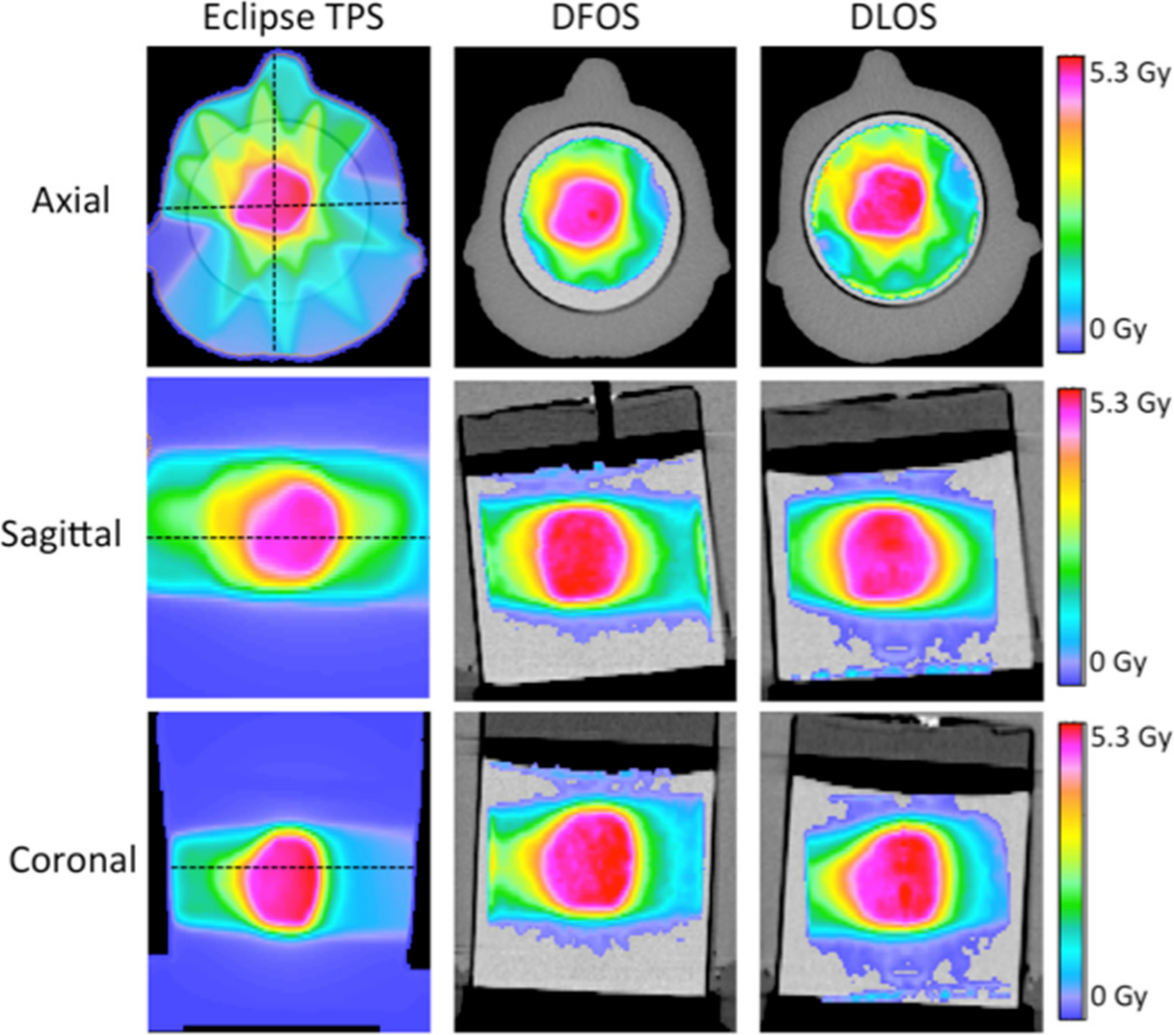
Treatment Plan 3—Results. Eclipse predicted dose (left), DFOS reconstructed dose (middle) and DLOS dose (right) for three orthogonal views in brain IMRT plan.

**Fig 8:**
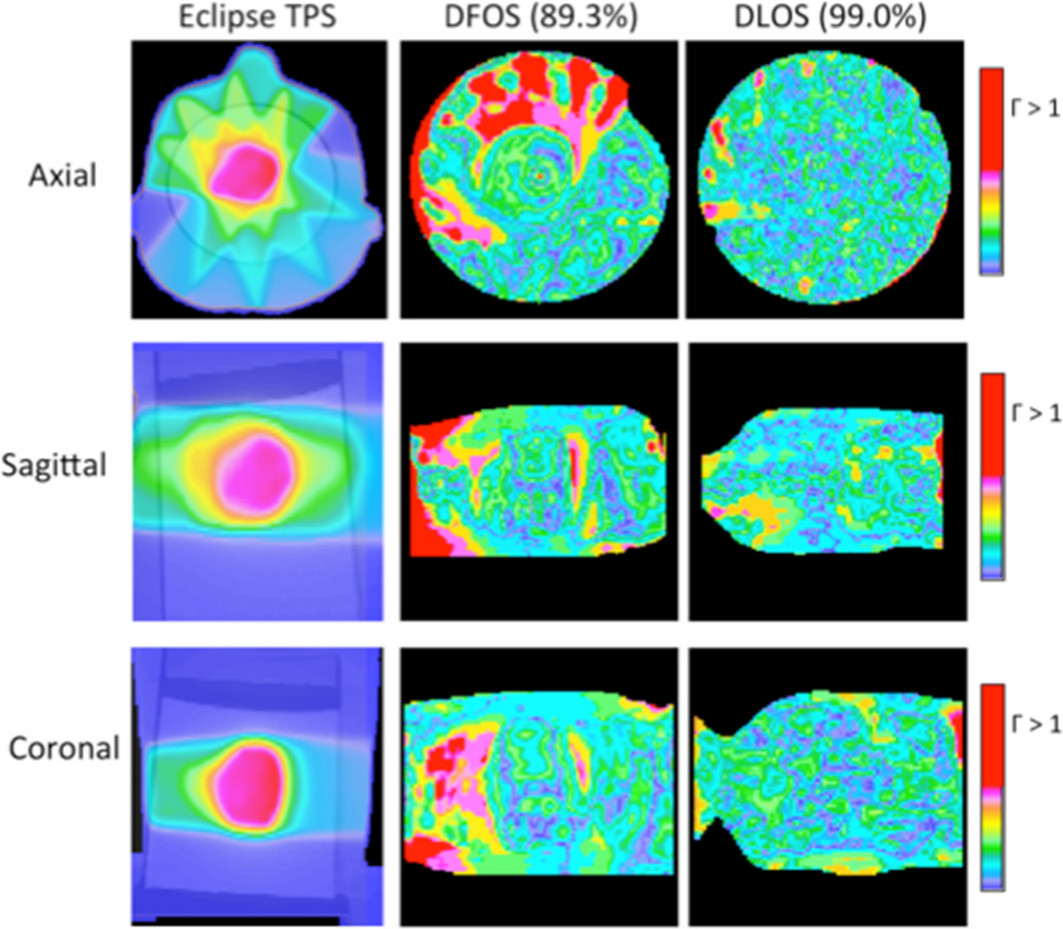
Treatment Plan 3—Gamma Maps. Three orthogonal views of 3%/3mm gamma maps for DFOS (column 2) and DLOS (right) systems, with passing rates. Eclipse dose views (left) for reference.

For the brain IMRT plan the DLOS measured dose distribution exhibited a gamma pass rate of 99.0%, substantially higher than the 89% achieved with DFOS. Both DLOS and DFOS gamma comparisons with the planned distribution fall inside the 88–90% recommendations of AAPM Task Group Report TG119 for a gamma criteria of 3%/3mm [27]. While the gamma rate drop between DLOS and DFOS indicates a loss of accuracy, these are promising first results from a prototype DFOS system. Figs 7 and 8 show orthogonal slices through measured dose and gamma distributions, respectively, for both systems. It can be seen in the gamma results in Fig 8 that the DLOS out-performs the DFOS in the regions of sharp dose gradients. It is possible that stray light contributes to dose reconstruction artifacts caused by some loss and degradation of the parallel ray geometry assumed by the reconstruction algorithm. This geometry is rigorously enforced in the telecentric systems (0.1degree tolerance). A detailed study of these subtle effects was beyond the scope of the current work, and is left to further investigation.

### DFOS Performance Summary

**Fig 9:**
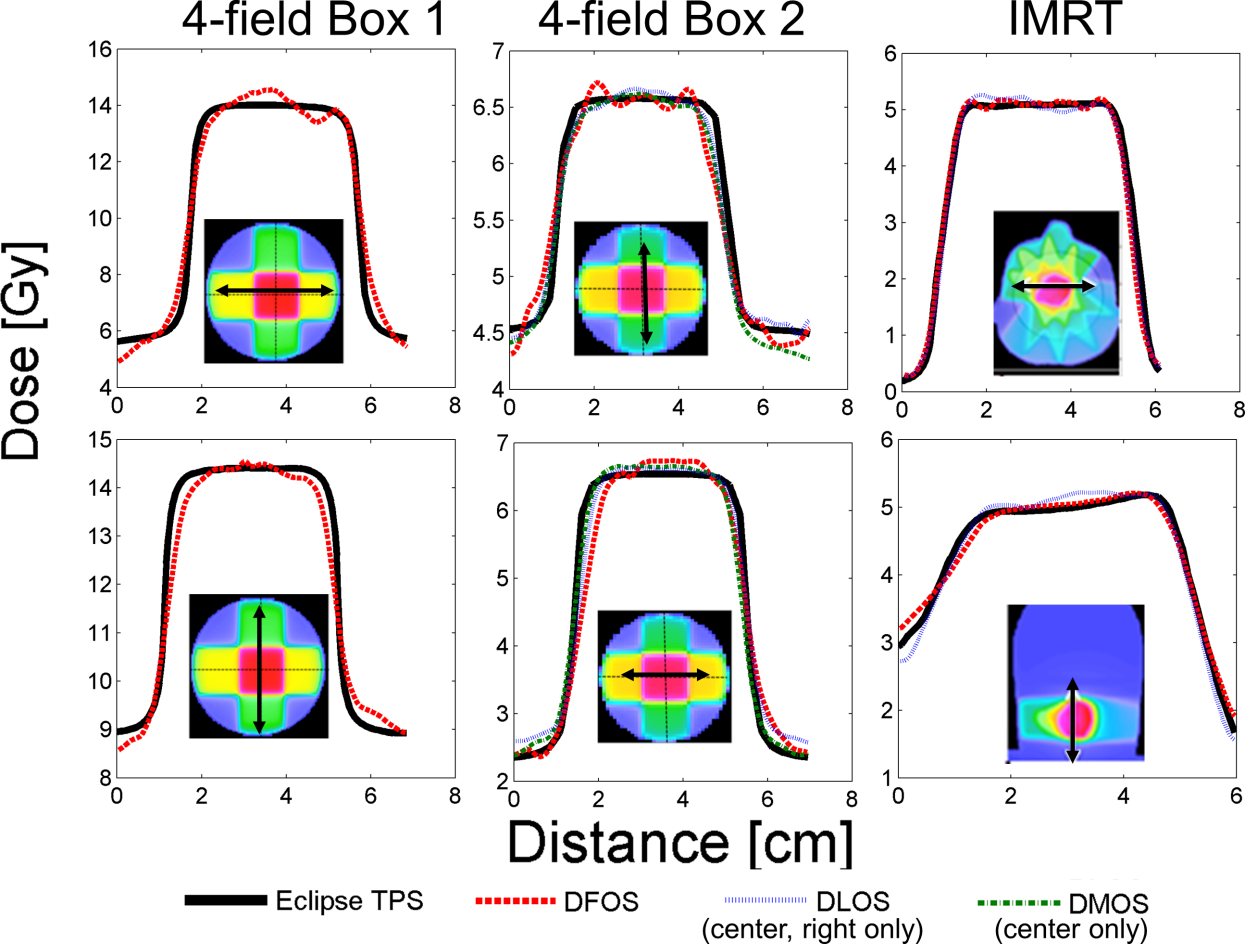
Dose Distribution Line Profiles. Representative line profiles from 3 dosimeters (irradiated with the 3 different dose distributions as indicated), each scanned with 3 optical-CT scanners. Elevated noise levels can be seen with the DFOS system.

Fig 9 shows line profiles through various orthogonal planes for each of the 3 treatment plans. Some notable observations–the DLOS system performed optimally for reproducing brain IMRT dose distributions, while the DMOS matched the DLOS in the second parallel-opposed treatment. DFOS line profiles show a substantial increase in noise when compared to DLOS and DMOS reconstructions. Although concurrent work showed the optimal dose-readout time for the DEA Presage formulation to be 3–24 hours post-irradiation, there is a significant rounding of sharp dose gradients shown with the all three systems in the second parallel-opposed treatment, which was readout ~20 hours post-irradiation. This is in comparison to a near perfect representation with the DLOS IMRT reconstruction, which features sharp dose gradients as well, but with readout performed 3 hours post-irradiation. Further investigation is needed to determine the reason for these inconsistencies.

Table 1 below summarizes all gamma pass rates for all treatments compared to Eclipse TPS prediction.

**Table 1:** Summary of gamma 3%/3mm pass rates for all irradiations.

Treatment	Dose Prescription	Gamma pass rate w.r.t. Eclipse TPS		
		DFOS	DLOS	DMOS
Parallel-opposed I	500MU / 250MU	89.3%	-	-
Parallel-opposed II	250MU / 125MU	92.2%	95.6%	96.8%
Brain IMRT	5-field (500 cGy)	89.3%	99.0%	-

**Fig 10:**
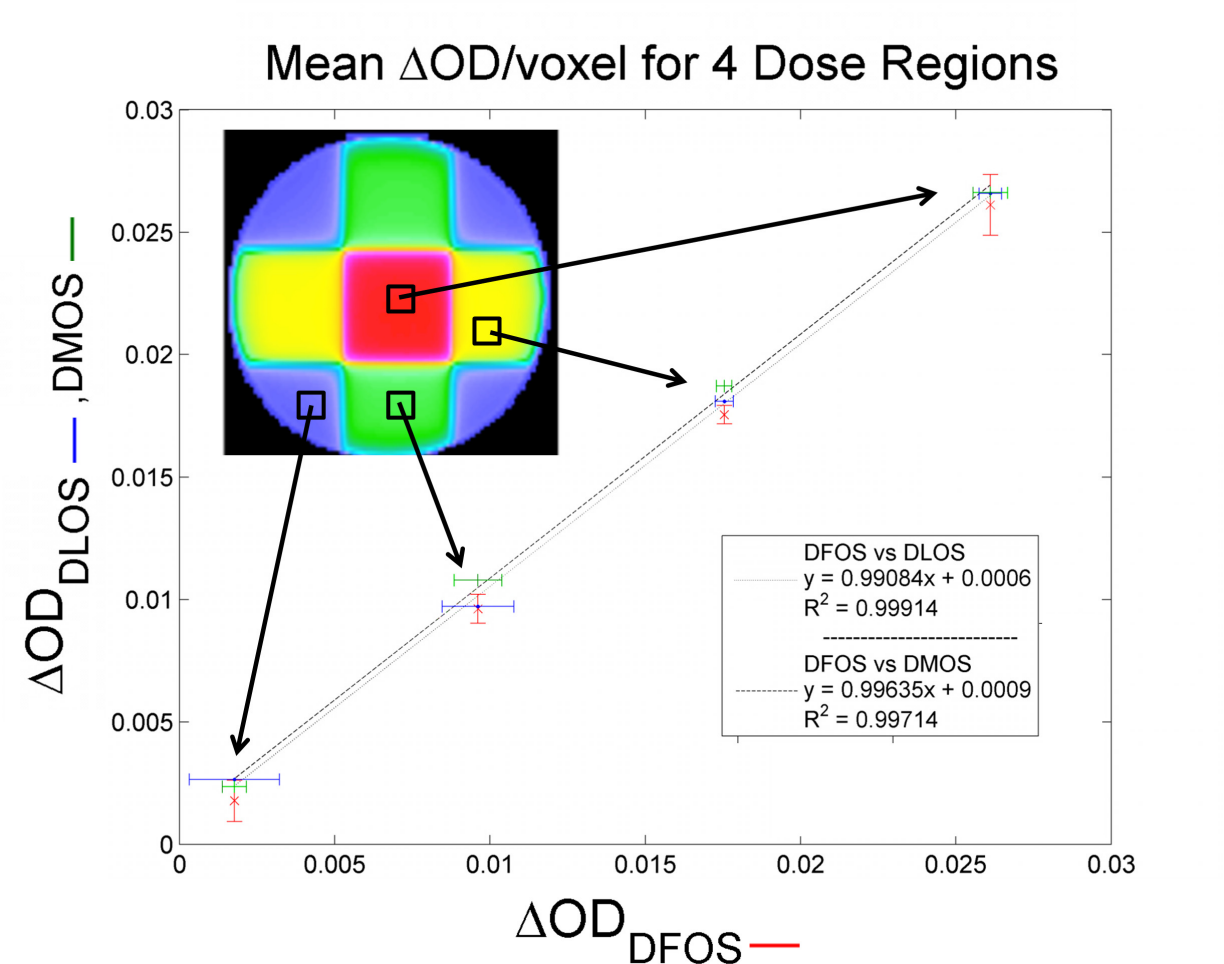
Comparison of All Three Systems. Mean OD change (ΔOD) for the 4 distinct dose regions within the 4-field box treatment. The DFOS system is compared to the DLOS (blue) and DMOS (green). Also shown are linear fits for the two gold-standard systems compared to DFOS. Error bars span 2 standard deviations (95% confidence) for all systems.

Consistency and noise levels of reconstructed ΔOD values obtained with the DFOS system were compared to DLOS and DMOS values in the second 4-field box treatment. Mean ΔOD and standard deviations were calculated for all three systems from ROIs within each of four distinct dose regions–high dose, medium dose, low dose, and no (scatter-only) dose regions. Fig 10 shows comparison of mean DFOS ΔOD values compared to DLOS and DMOS, with 2 standard deviations shown. Linear fits between the DFOS and gold-standard systems show good agreement with slopes of 0.991 and 0.996 for the DLOS and DMOS, respectively. Noise levels were further evaluated by comparing the coefficient of variation (COV) for the 3 regions within the treatment beams for all three systems. The no dose/scatter only region was ignored due to high variations in the very low scatter dose. Noise levels were low for all three with a maximum COV of 3.1%. The DFOS system showed a slight noise increase compared to the other systems with 1.1%-3.1% COV, compared to 0.8%-2.0% and 0.7%-2.4% for the DLOS and DFOS, respectively. COVs are summarized in Table 2.

**Table 2:** Coefficient of variation for mean ΔOD values for all systems, calculated for the 3 treated dose regions within the second 4-field box treatment plan.

System	Coefficient of Variation		
	Low	Medium	High
**DFOS**	3.1%	1.1%	2.4%
DLOS	2.0%	0.8%	1.0%
DMOS	2.4%	0.7%	1.0%

## Conclusion

This work attempts to improve the practicality of telecentric optical-CT systems by using a solid-tank to reduce the required volumes of refractively matched fluid by up to 98% (with associated fluid handling and maintenance savings) and reducing the cost of the lenses by up to 90%. Utilization of a solid-tank has benefits in reducing fluid and fluid management overheads, but our data show that ring artifacts and overall noise are increased with the use if a solid tank and Fresnel lenses. The latter point is in contrast with our hypothesis that minimizing refractive index-matching fluid would lead to improved flood field corrections, given that impurities that in the fluid baths would decrease due to decreased fluid volume. Our data also shows that the DFOS incorporation of Fresnel lenses degraded image quality through Moiré and loss of telecentricity, rendering current overall performance substantially inferior to the gold standard telecentric systems. Although initial solid tank and Fresnel lens results show some level of quantitative accuracy, further work is required to improve the quality of the system for optical-CT 3D dosimetry. This work highlights both the advantages (convenience and cost) and disadvantages (loss of accuracy and increased imaging artifacts) of the current prototype DFOS system. Taken together the results show promising potential for DFOS but further optimization and improvement in tank design and image artifact suppression is required to achieve similar capability to DLOS system.

## Supporting Information

S1 File MATLAB data set corresponding to 4-field box treatment 1. (ZIP)

S2 File MATLAB data set corresponding to 4-field box treatment 2. (ZIP)

S3 File MATLAB data set corresponding to IMRT treatment 1. (ZIP)
